# Knockout of Endothelial Cell-Derived Endothelin-1 Attenuates Skin Fibrosis but Accelerates Cutaneous Wound Healing

**DOI:** 10.1371/journal.pone.0097972

**Published:** 2014-05-22

**Authors:** Katsunari Makino, Masatoshi Jinnin, Jun Aoi, Ikko Kajihara, Takamitsu Makino, Satoshi Fukushima, Keisuke Sakai, Kazuhiko Nakayama, Noriaki Emoto, Masashi Yanagisawa, Hironobu Ihn

**Affiliations:** 1 Department of Dermatology and Plastic Surgery, Faculty of Life Sciences, Kumamoto University, Kumamoto, Japan; 2 Clinical Pharmacy, Kobe Pharmaceutical University, Kobe, Japan; 3 Department of Molecular Genetics and Howard Hughes Medical Institute, University of Texas Southwestern Medical Center, Dallas, Texas, United States of America; 4 International Institute for Integrative Sleep Medicine (WPI-IIIS) and Center for Behavioral Molecular Genetics, University of Tsukuba, Tsukuba, Japan; French National Centre for Scientific Research, France

## Abstract

Endothelin (ET)-1 is known for the most potent vasoconstrictive peptide that is released mainly from endothelial cells. Several studies have reported ET-1 signaling is involved in the process of wound healing or fibrosis as well as vasodilation. However, little is known about the role of ET-1 in these processes. To clarify its mechanism, we compared skin fibrogenesis and wound repair between vascular endothelial cell-specific ET-1 knockout mice and their wild-type littermates. Bleomycin-injected fibrotic skin of the knockout mice showed significantly decreased skin thickness and collagen content compared to that of wild-type mice, indicating that bleomycin-induced skin fibrosis is attenuated in the knockout mice. The mRNA levels of transforming growth factor (TGF)-β were decreased in the bleomycin-treated skin of ET-1 knockout mice. On the other hand, skin wound healing was accelerated in ET-1 knockout mice, which was indicated by earlier granulation tissue reduction and re-epithelialization in these mice. The mRNA levels of TGF-β, tumor necrosis factor (TNF)-α and connective tissue growth factor (CTGF) were reduced in the wound of ET-1 knockout mice. In endothelial ET-1 knockout mouse, the expression of TNF-α, CTGF and TGF-β was down-regulated. Bosentan, an antagonist of dual ET receptors, is known to attenuate skin fibrosis and accelerate wound healing in systemic sclerosis, and such contradictory effect may be mediated by above molecules. The endothelial cell-derived ET-1 is the potent therapeutic target in fibrosis or wound healing, and investigations of the overall regulatory mechanisms of these pathological conditions by ET-1 may lead to a new therapeutic approach.

## Introduction

Endothelin-1 (ET)-1, one of the three members of ET family, is known as the most potent vasoconstrictive peptide. The molecule is released mostly from endothelial cells [1], [2], and its biological actions are mediated by two different receptors, ET_A_ and ET_B_ [3]. In addition to its effect as a vasoconstrictor, ET-1 can stimulate smooth muscle cell proliferation [4]. Furthermore, the molecule induces the expression of several proto-oncogenes such as c-myc or c-fos [5]. Through such diverse biological activities, ET-1 signal is thought to play central roles in several pathological conditions including pulmonary hypertension.

ET-1 is also found to induce collagen expression in cultured fibroblasts of heart, skin or kidney [6]–[8]. In addition, bosentan, an antagonist of dual ET receptors, reduced the number of digital ulcers in patient with systemic sclerosis, an autoimuune disorder characterized by tissue fibrosis of the skin and internal organs [9]. These results indicate ET-1 signal also has a profibrotic effect *in vitro* and *in vivo*. On the other hand, several researches suggested the drug improves skin fibrosis of systemic sclerosis [10]. Thus, detailed mechanism of ET-1 effects on fibrosis and wound heal is still to be clarified.

Homozygous deletion of ET-1 in mice shows early postnatal lethality caused by craniofacial abnormalities [11]. Therefore, considering that the major source of ET-1 is endothelial cells, we utilized endothelial cell-specific ET-1 knockdown mice for analyzing the mechanism of ET-1 involvement in the skin fibrosis and wound healing.

## Materials and Methods

### Ethics Statement

All animal experimental protocols in this study were approved by the Committee on the Animal Research at Kumamoto University (Permit Number: 24–150). All efforts were made to minimize suffering.

### Mice

Eight-week-old heterozygous ET-1^f/f^; Tie-2-Cre (+) mice and control ET-1^f/f^; Tie-2-Cre (−) littermates (WT, wild-type mice) were used for experiments. ET-1^f/f^; Tie-2-Cre (+) mice were generated as described [12], [13]. In brief, mice strains with ET-1 exon 2 flanked by loxP sites were prepared. Tie2-Cre transgenic mice expressing Cre recombinase in a pan-endothelial fashion were utilized for vascular endothelium-specific targeting. By breeding those mice, genetically modified mice depleting the preproET-1 gene specifically in endothelial cells were obtained. The mice were housed in a specific pathogen-free and temperature-controlled environment with a 12-hour light/dark cycle and were fed a standard diet and water *ad libitum*. They did not display any evidence of infection throughout the study. In all experiment, the sex ratio was same between groups.

### Bleomycin-induced skin fibrosis in mice

Bleomycin treatment was performed as previously reported [14]. In brief, bleomycin (Nippon Kayaku) was dissolved in phosphate buffered saline (PBS) at a concentration of 1 mg/ml and sterilized by filtration. Bleomycin (100 µl) was injected intradermally into the shaved back of the 8-week-old mice daily for 4 weeks. The back skin was removed on day after final bleomycin injection.

### Wound healing experiment

Under the local anesthesia, one full-thickness excisional wound was generated on the dorsal skin using an 8 mm diameter dermal punch. After wounded, mice were caged individually. The wound and surrounding tissue were collected at days 0, 3, 7, 12 post-wounding.

### Staining

Skin samples were paraffin-embedded, and sections were dewaxed in xylene and rehydrated in graded alcohols. Haematoxylin and eosin (HE) staining or Masson's trichrome staining was performed as described previously [15].

For immunostaining, antigens were retrieved by incubation with proteinase K (DAKO) for 6 minutes. Endogenous peroxidase activity was inhibited, after which sections were blocked with 3% bovine serum albumin (BSA, Sigma) for 20 minutes and then reacted with the primary antibodies for ET-1 (1∶250, Peninsula Laboratories), myeloperoxidase (1∶100, Thermo), F4/80 (1∶100, Abcam), or CD3 (1∶100, Serotec) overnight at 4°C. After excess antibody was washed out with PBS, sections were incubated with appropriate HRP-labeled secondary antibody (Nichirei) for 60 minutes at 20°C. The reaction was visualized by diaminobenzidine substrate system (Dojin). Slides were counterstained with Mayer's haematoxylin, and examined under a light microscope (OLYMPUS).

### Immunofluorescence

Paraffin sections were deparaffinized in xylen and rehydrated in a graded ethanol series. Antigens were retrieved by incubation with proteinase K for 5 minutes. The slides were blocked in 3% BSA for 60 minutes. As the primary antibodies, rabbit anti-ET-1 polyclonal antibody (1∶250, Peninsula Laboratories) and Isolectin IB4 Alexa Fluor 488 dye conjugate (1∶200, Invitrogen) were applied to the sections overnight at 4°C [16], [17]. After excess antibody was washed out with PBS, a species-matched Alexa 546-labeled secondary antibody (Invitrogen) was added. After 1 hour at room temperature, sections were washed and mounted with VECTASHIELD mounting medium (Vector). Fluorescence images of Alexa 488 and Alexa 546 were recorded with Biozero BZ-8000 fluorescence microscope (KEYENCE).

### Measurement of collagen contents in tissue sections

Collagen contents in paraffin-embedded skin sections were determined using quantitative micro-assay kit (Chondrex) following the manufacturer's instructions. This method is based on the selective binding of Sirius Red and Fast Green to collagens and non-collagen proteins, respectively [18]. Briefly, 10 µm-thick sections were deparaffinized, and stained with Sirius Red and Fast Green. The color in the tissue sections was eluted by dye extraction solution. Absorbance was measured in a spectrophotometer at OD540 (for Sirius Red) and OD605 (for Fast Green), respectively. The amounts of collagen and non-collagenous proteins in each section were determined by interpolation from a standard curve.

### RNA isolation and real-time PCR

Total RNAs were extracted from skin samples using ISOGEN (Nippon Gene). First-strand cDNA was synthesized by PrimeScript RT reagent Kit (Takara). Quantitative real-time PCR was performed on Takara Thermal Cycler Dice (TP800^®^) using primers and templates mixed with the SYBR Premix Ex Taq II Kit (Takara). Primer sets for transforming growth factor (TGF)-β1, TGF-β3, α2 (I) collagen, connective tissue growth factor (CTGF), Tumor necrosis factor (TNF)-α, E-selectin and glyceraldehyde 3-phosphate dehydrogenase (GAPDH) were purchased from Takara. DNA was amplified for 40 cycles of denaturation for 5 seconds at 95°C and annealing for 30 seconds at 60°C. Data generated from each PCR reaction were analyzed using Thermal Cycler Dice Real Time System ver 2.10B (Takara). The relative fold change of each gene was calculated by standard curve method. Transcript level of gene of interest was normalized to that of GAPDH in the same sample.

### Statistical analysis

The statistical analysis was carried out with Mann-Whitney U test for the comparison of medians. All analyses were performed with Statcel3 software (OMS). *P* values <0.05 were considered to be significant.

## Results

### Expression of ET-1 in the skin of ET-1^f/f^; Tie-2-Cre (+) mice

**Figure 1:**
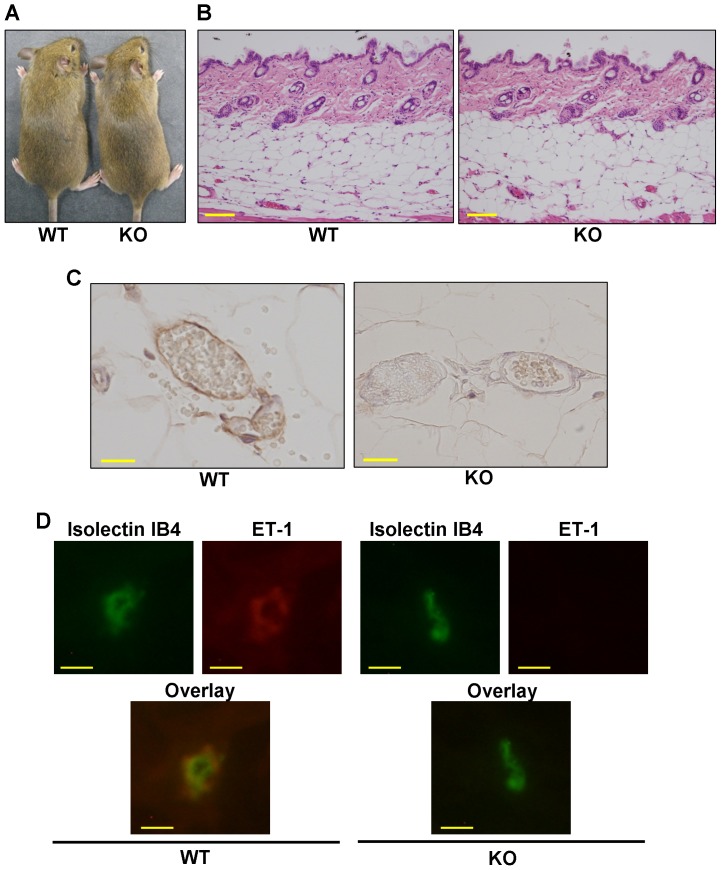
Comparison of the ET-1 expression between wild-type and ET-1^f/f^; Tie-2-Cre (+) mice skins. (A) Gross comparison of a wild-type (WT) and an ET-1^f/f^; Tie-2-Cre (+) (KO) mouse at 8 weeks. (B) Representative haematoxylin and eosin (HE) staining of skin section from a wild-type (WT) and an ET-1^f/f^; Tie-2-Cre (+) (KO) mouse. (C) Representative ET-1 staining of subcutaneous blood vessels in a wild-type (WT) and an ET-1^f/f^; Tie-2-Cre (+) (KO) mouse. Scale bar = 20 µm. (D) Dermal vessels of wild-type (WT) and ET-1^f/f^; Tie-2-Cre (+) (KO) mice were stained with antibodies against isolectin IB4 (green) and ET-1 (red). Scale bar = 10 µm.

ET-1 whole-body knockout mice were dead shortly after birth, while the heterozygote ET-1^f/f^; Tie2-Cre (+) mice were born with no defects and grew up without apparent abnormalities [11], [12]. As an initial experiment, we compared a skin phenotype of ET-1^f/f^; Tie-2-Cre (+) mice and WT mice. The macroscopic appearance was similar between these mice (Fig. 1A). Additionally, the microscopic skin appearances of ET-1^f/f^; Tie-2-Cre (+) mice were not different from those of WT mice (Fig. 1B).

We then confirmed the expression of ET-1 peptide in vascular endothelial cells of the dorsal skin. By immunohistochemical staining, ET-1 was observed in vessels of WT mice, but not in ET-1^f/f^; Tie-2-Cre (+) mice (Fig. 1C). Similarly, immunofluorescence revealed co-staining of ET-1 and Isolectin IB4, a marker of endothelial cells, in WT mice (Fig. 1D left), but not in ET-1^f/f^; Tie-2-Cre (+) mice (Fig. 1D right). Therefore, we confirmed that ET-1 was successfully knocked-out in the skin blood vessels of ET-1^f/f^; Tie-2-Cre (+) mice.

### Bleomycin-induced skin fibrosis in ET-1^f/f^; Tie-2-Cre (+)

**Figure 2:**
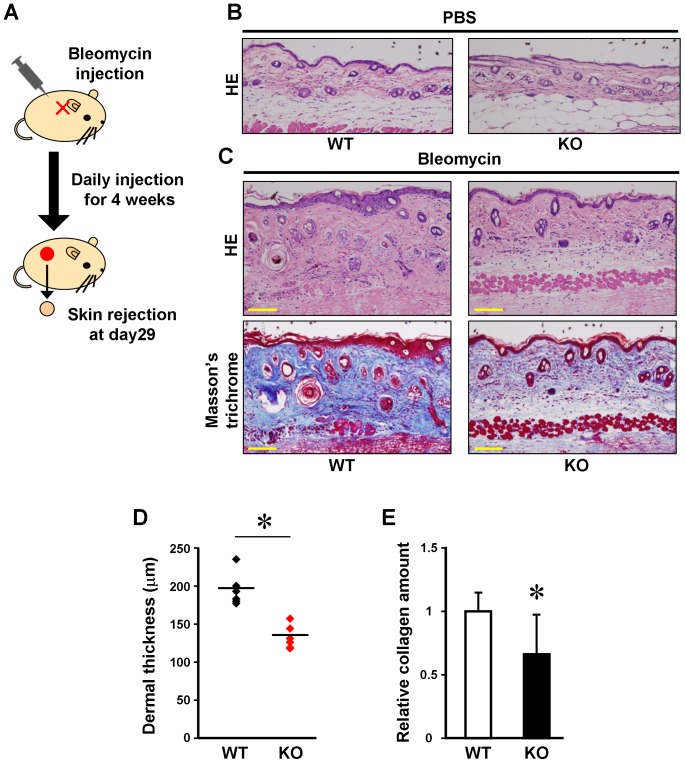
Bleomycin-induced skin fibrosis in wild-type and ET-1^f/f^; Tie-2-Cre (+) mice. (A) The protocol for Figure 2B and 2C is shown. Bleomycin or PBS was locally injected in the back of the wild-type (WT) and ET-1^f/f^; Tie-2-Cre (+) (KO) mice daily for 4 weeks. The back skin was obtained on day 29. (B) Hematoxylin and eosin (HE) staining of PBS-treated mice skin. WT; wild-type, KO; ET-1^f/f^; Tie-2-Cre (+). Scale bar = 100 µm. (C) HE (upper panels) and Masson's trichrome staining (lower panels) of bleomycin-treated mice skin. WT; wild-type, KO; ET-1^f/f^; Tie-2-Cre (+). Scale bar = 100 µm. (D) Dermal thickness of the wild-type (WT) and ET-1^f/f^; Tie-2-Cre (+) (KO) mice was evaluated by measuring the distance between the epidermal-dermal junction and the dermal-fat junction in HE sections under 100-folds magnification. Data are shown on the ordinate (n = 6). Bars show means. *P<0.05. (E) Relative collagen contents of paraffin-embedded sections from the wild-type (WT) and ET-1^f/f^; Tie-2-Cre (+) (KO) mice were determined as described in “Materials and Methods” (n = 6). *P<0.05.

Based on above results, we first determined the possibility that the knockout of endothelial cell-derived ET-1 affects the onset of skin fibrosis. As shown in Fig. 2A, skin fibrosis was induced on the back of mice skin by intradermal bleomycin injection daily for 4 weeks. Then, the back skin was removed one day after the final bleomycin injection.

When PBS was injected in back skin as the control, the skin structure and dermal thickness did not differ between ET-1^f/f^; Tie-2-Cre (+) and WT mice (Fig. 2B). In WT mice, the skin injected with bleomycin showed dermal fibrosis with thickened dermis, increased number of collagen bundles and strong Masson's trichrome staining (Fig. 2C left). On the other hand, the bleomycin-treated skin of ET-1^f/f^; Tie-2-Cre (+) mice showed the thinner dermal thickness and weaker Masson's trichrome staining in the dermis, (Fig. 2C right), as compared to those of WT mice. We confirmed such improvement of bleomycin-induced dermal thickening in ET-1^f/f^; Tie-2-Cre (+) mice was statistically significant (Fig. 2D). Consistently, the collagen contents in bleomycin-treated skin of ET-1^f/f^; Tie-2-Cre (+) mice was significantly lower than those in WT mice (Fig. 2E). Therefore, ET-1 may positively contribute to the development of skin fibrosis.

### Infiltrating inflammatory cell profile and ECM-related gene expression in the skin of bleomycin-treated ET-1^f/f^; Tie-2-Cre (+) mice

**Figure 3:**
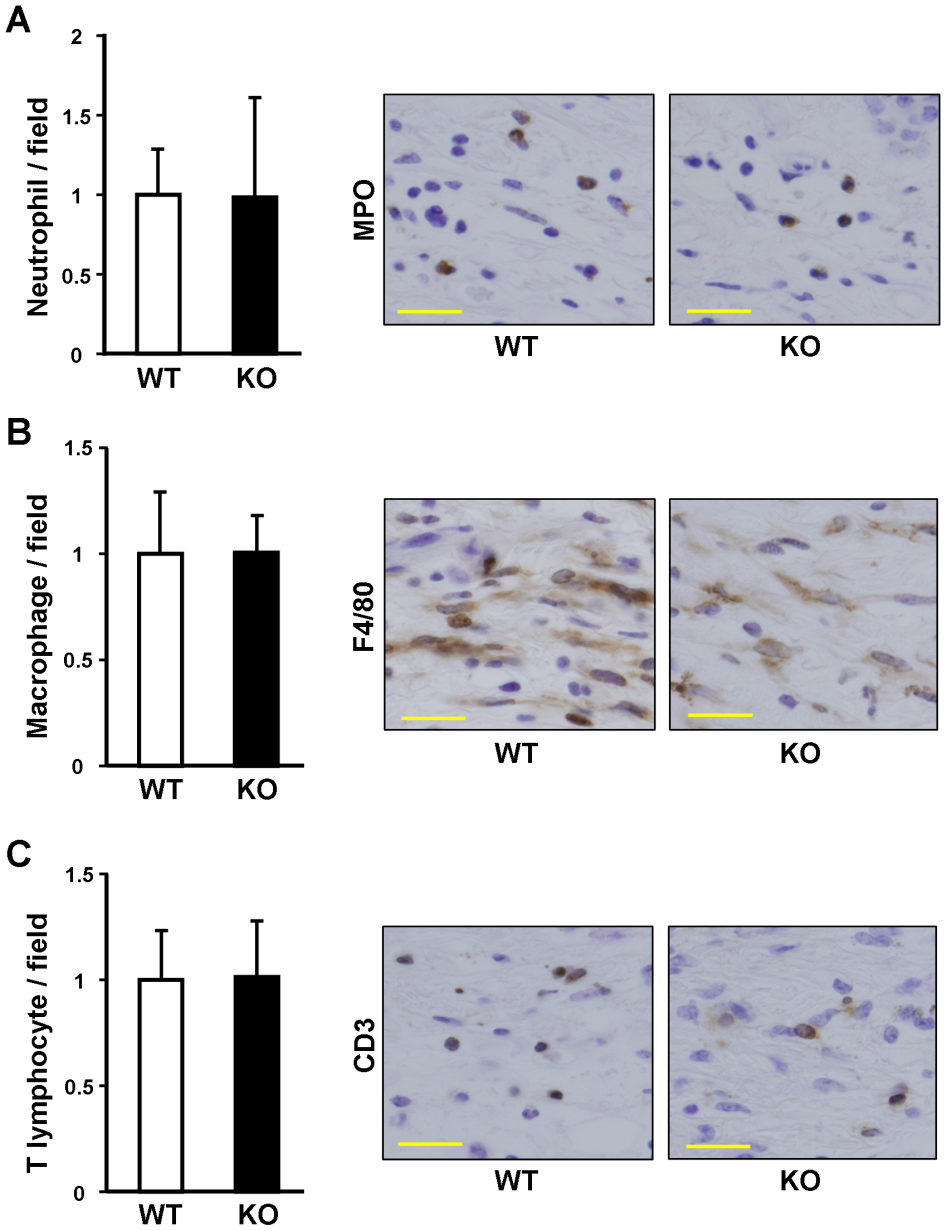
Infiltrating cells in the bleomycin-treated skin of the wild-type (WT) and ET-1^f/f^; Tie-2-Cre (+) (KO) mice. Myeloperoxidase (A), F4/80 (B) and CD3 (C) were stained. Positive cells were counted in five random high-power fields (0.06 mm^2^, magnification, ×400). Data were expressed as the mean ± SD of six independent counts (left panel). The representative results of immunostaining for myeloperoxidase, F4/80 and CD3 are shown (right panel).

To clarify the mechanism by which endothelial cell-derived ET-1 mediates cutaneous fibrosis, we then compared the number of inflammatory cells in bleomycin-induced fibrotic skin between ET-1^f/f^; Tie-2-Cre (+) mice and WT mice. Myeloperoxidase-positive neutrophils, F4/80-positive macrophages, or CD3-positive T cells were counted in immunohistochemical staining sections. No differences between these mice were seen in the number of neutrophils (Fig. 3A), macrophages (Fig. 3B) and T cells (Fig. 3C).

**Figure 4:**
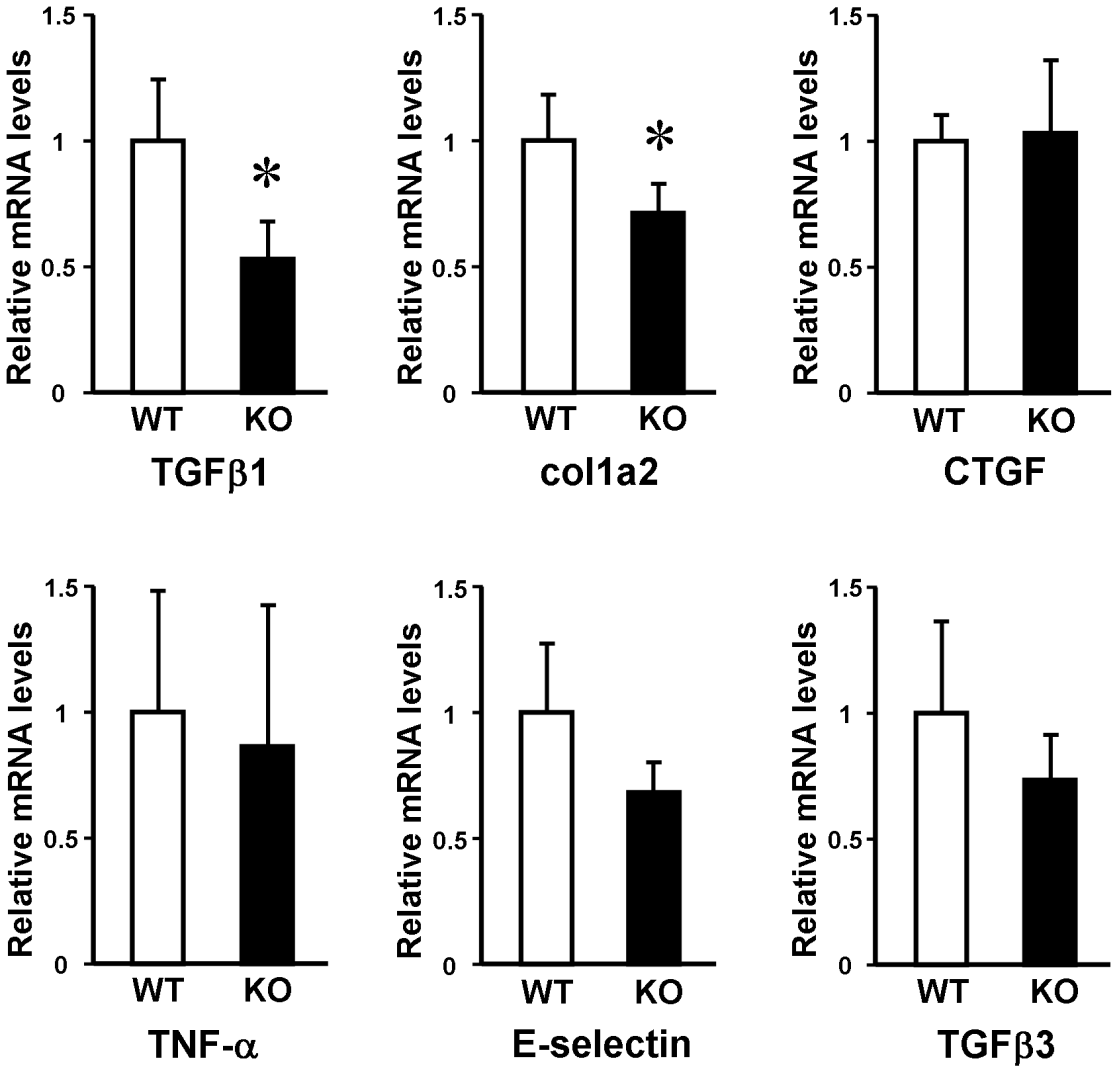
Cytokine expression in the bleomycin-treated skin from the wild-type (WT) and ET-1^f/f^; Tie-2-Cre (+) (KO) mice. Total RNA was extracted from the skin, and the mRNA expression levels of indicated cytokines were determined by real-time PCR. Data are expressed as the mean ± SD of six independent experiments. *P<0.05 as compared with the value in WT mice (1.0).

In addition, we compared the expression of various ECM-related molecules in the bleomycin-treated skin of WT mice and ET-1^f/f^; Tie-2-Cre (+) mice by quantitative real-time PCR (Fig. 4). ET-1^f/f^; Tie-2-Cre (+) mice skin showed significantly decreased mRNA levels of TGF-β1 and α2 (I) collagen relative to WT mice. In contrast, the mRNA levels of TGF-β3, CTGF, TNF-α and E-selectin were not different between these mice. Before the treatment, these levels in the dorsal skin were similar between ET-1^f/f^; Tie-2-Cre (+) and WT mice (data not shown). Thus, our results indicated that decreased mRNA levels of TGF-β1 and α2 (I) collagen cause the attenuated skin fibrosis in ET-1^f/f^; Tie-2-Cre (+) mice.

### Wound closure, granulation tissue formation, and re-epithelialization in ET-1^f/f^; Tie-2-Cre (+) mice

**Figure 5:**
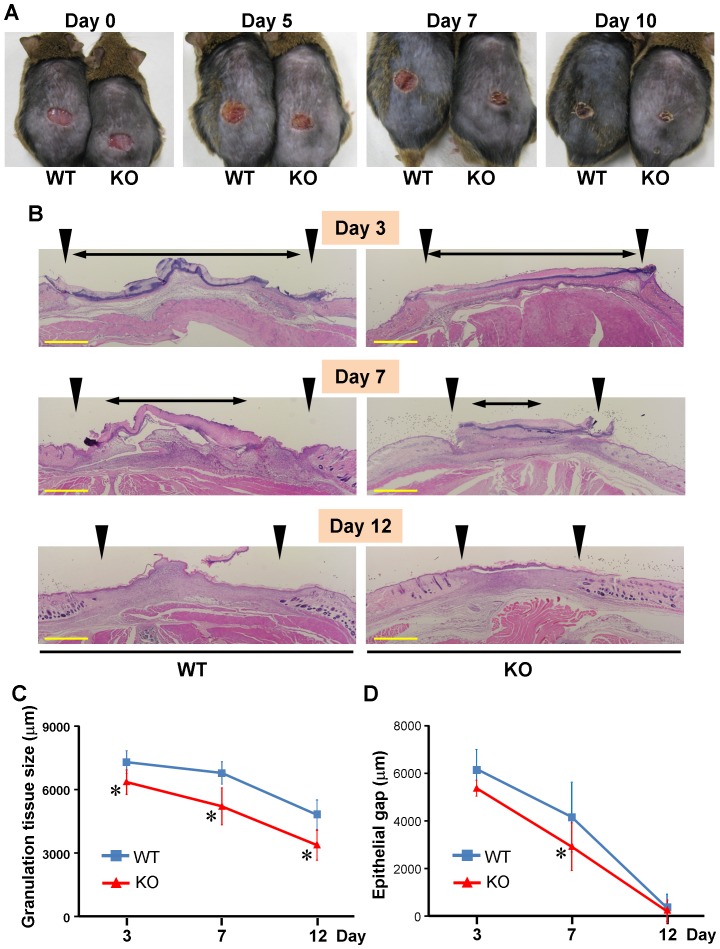
Cutaneous wound healing in WT and ET-1^f/f^; Tie-2-Cre (+) mice. (A) Representative wound closure in wild-type (WT) and ET-1^f/f^; Tie-2-Cre (+) (KO) mice at days 0, 5, 7, 10 post-wounding. (B) Hematoxylin-Eosin (HE) staining of wound tissues derived from wild-type (WT) and ET-1^f/f^; Tie-2-Cre (+) (KO) mice at days 3, 7, 12 post-wounding. Arrow heads indicated bilateral edges of wound granulation tissue. Double-headed arrows indicated the distance between the leading edges of wounded epidermis. The one representative result is shown. Scale bar = 1000 µm. (C) Measurements of granulation tissue size (the distance between the arrow heads) in the wild-type (WT) and ET-1^f/f^; Tie-2-Cre (+) (KO) mice at days 3, 7, 12 post-wounding. Data are expressed as the mean ± SD of six independent experiments. *P<0.05 as compared with the value in WT mice. (D) Measurements of epithelial gap (the distance of double-headed arrows) in the wild-type (WT) and ET-1^f/f^; Tie-2-Cre (+) (KO) mice at days 3, 7, 12 post-wounding. Data are expressed as the mean ± SD of six independent experiments. *P<0.05 as compared with the value in WT mice.

Next, we investigate the role of vascular endothelial cell-derived ET-1 in cutaneous wound repair. A full-thickness 8 mm excisional wound was made in the dorsal skin of ET-1^f/f^; Tie-2-Cre (+) and WT mice, and they were examined for up to 12 days after wounded. In macroscopically, ET-1^f/f^; Tie-2-Cre (+) mice showed enhanced wound healing than WT mice (Fig. 5A).

Then, wound and surrounding tissue were collected at days 3, 7 and 12 post-wounding, and were used to evaluate granulation tissue area (the distance between bilateral edges of granulation tissue consisted of newly formed capillaries, fibroblasts and macrophages) and epithelial gap (the distance between the leading edges of wounded epidermis) in HE stained section (Fig. 5B). The granulation tissue area was significantly smaller in ET-1^f/f^; Tie-2-Cre (+) mice than in WT mice at day 3, which continued till day 12 (Fig. 5C). Similarly, the epithelial gap was significantly shorter in ET-1^f/f^; Tie-2-Cre (+) mice than in WT mice at days 7 (Fig. 5D). Thus, the accelerating wound closure in ET-1^f/f^; Tie-2-Cre (+) mice was also confirmed microscopically.

**Figure 6:**
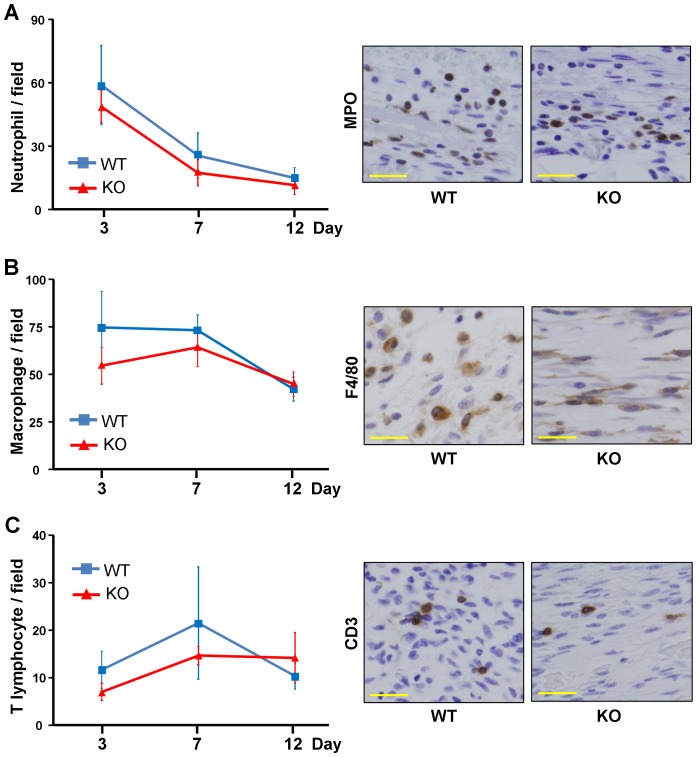
Infiltrating cells in the wounding bed at day 3, 7 and 12 of the wild-type (WT) and ET-1^f/f^; Tie-2-Cre (+) (KO) mice. Myeloperoxidase (A), F4/80 (B) and CD3 (C) were stained. Positive cells were counted in five random high-power fields (0.06 mm^2^, magnification, ×400). Data were expressed as the mean ± SD of six independent counts (left panel). The representative results of immunostaining for myeloperoxidase, F4/80 and CD3 are shown (right panel).

When the myeloperoxidase-positive neutrophils, F4/80-positive macrophages, or CD3-positive T cells were counted by immunostaining, the number of these cells tended to be slightly decreased in the wounding bed at days 3, 7, and 12 in ET-1^f/f^; Tie-2-Cre (+) mice (Fig. 6A-C), but not statistically significant.

**Figure 7:**
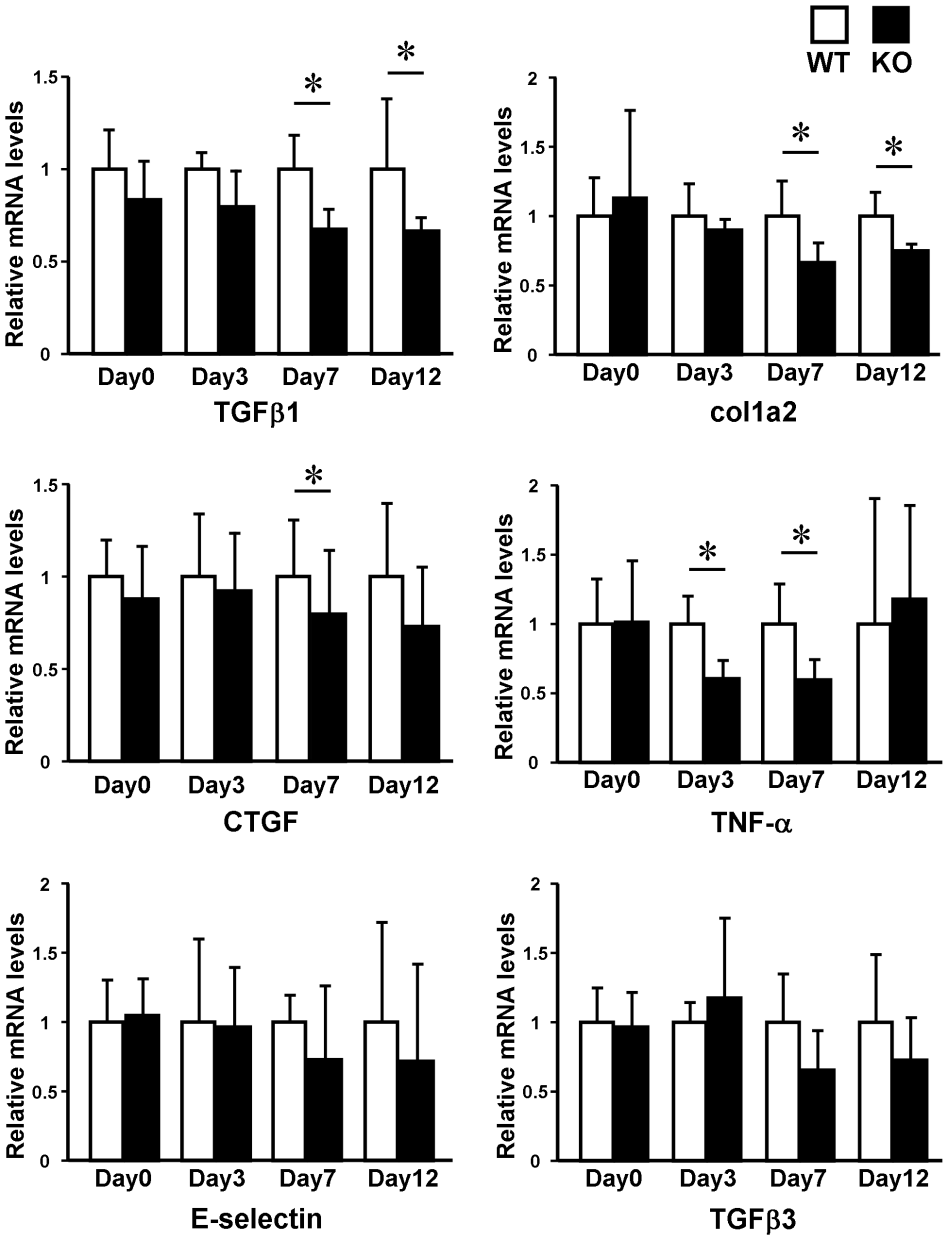
Cytokine expression in the wounding bed at day 3, 7 and 12 of the wild-type (WT) and ET-1^f/f^; Tie-2-Cre (+) (KO) mice. Total RNA was extracted from the skin, and the mRNA expression levels of indicated cytokines were determined by real-time PCR. Data are expressed as the mean ± SD of six independent experiments. *P<0.05 as compared with the value in WT mice (1.0).

In addition, we compared the expression of ECM molecules in the wounding bed between these mice by quantitative real-time PCR (Fig. 7). At day 3 after wounding, ET-1^f/f^; Tie-2-Cre (+) mice skin showed significantly decreased mRNA levels of TNF-α relative to WT mice. Furthermore, at day 7 after wounding, the mRNA levels of TNF-α, TGF-β1, CTGF, and α2 (I) collagen were down-regulated in ET-1^f/f^; Tie-2-Cre (+) mice in comparison with those in WT mice. The decreased mRNA levels of TGF-β1 and α2 (I) collagen in ET-1^f/f^; Tie-2-Cre (+) mice continued till day 12 after wounding.

## Discussion

This study is the first, to our knowledge, to report the role of endothelial ET-1 in skin fibrosis and wound repair via regulating the expression of cytokines and ECM, using *in vivo* model.

The skin fibrosis induced by bleomycin injection in mice is known for a murine model of systemic sclerosis [14], [19]. In this mouse model, inflammation cell such as T cells and macrophages are seen in the fibrotic lesion [20]. In addition, the development of bleomycin-induced skin fibrosis is accompanied by evidence of activation of TGF-β signaling [20]. We demonstrated that bleomycin-induced skin fibrosis was inhibited in endothelial cell-specific ET-1 knockout mice. Furthermore, we showed that the mRNA levels of TGF-β and α2 (I) collagen were decreased in bleomycin-treated skin of ET-1 knockout mice. On the other hand, there were no apparent differences in the number of inflammatory cells between endothelial ET-1 knockout mice and WT mice. Accordingly, the attenuated skin fibrosis in endothelial ET-1 knockout mice is likely to result from the lower TGF-β levels and subsequent lower collagen expression. Although previous studies have shown that ET-1 expression is potently regulated by TGF-β in endothelial cells and fibroblasts [21]–[23], our study first demonstrated ET-1 can also regulate TGF-β expression *in vivo*.

In the current study, we also observed that cutaneous wound healing in endothelial cell-specific ET-1 knockout mice was accelerated than in WT mice. Wound healing in the skin is a complex process including inflammation phase, new tissue formation phase, and tissue remodeling phase [24]. The interactions among the blood vessels, epidermis, leukocytes, dermal fibroblasts, ECM, various growth factors and cytokines are involved in each phase through controlling the replacement of granulation tissue by collagenous tissue and re-epithelialization [25], [26]. The earlier reduction of granulation tissue and increased re-epithelialization were seen in ET-1 knockout mice. However, there were no significant differences in inflammatory cell numbers between endothelial ET-1 knockout mouse and WT mice. On the other hand, the mRNA level of TNF-α, a proinflammatory cytokine, was significantly lower in early wound tissue of endothelial ET-1 knockout mice than that of WT mice. According to the previous literatures, the inhibition of TNF-α may have therapeutic value for refractory wound [27]. For example, wound healing in mouse is impaired by TNF-α up-regulation via the dysregulated inflammation and apoptosis [28]. Wound healing in TNF-α receptor-deficient mice was accelerated by reducing leukocyte infiltration [29]. Hence, the lower level of TNF-α in early wound of endothelial ET-1 knockout mouse may be one of the mechanisms for accelerating wound healing via affecting inflammatory phase. On the other hand, as seen in bleomycin-induced fibrosis model, the mRNA levels of TGF-β as well as α2 (I) collagen and CTGF were reduced in the wound of endothelial ET-1 knockout mice. Although TGF-β is an inducer of fibrosis via the synthesis of collagen and CTGF, previous studies have demonstrated that TGF-β may express adverse effects on the wound healing [30]–[34]. Furthermore, it is known that the early-gestation fetus has the prominent ability to cure skin wounds earlier without scarring, due to the lower levels of TGF-β [35], [36]. Thus, reduced TGF-β expression may be another cause of the early would healing in endothelial ET-1 knockout mice.

**Figure 8:**
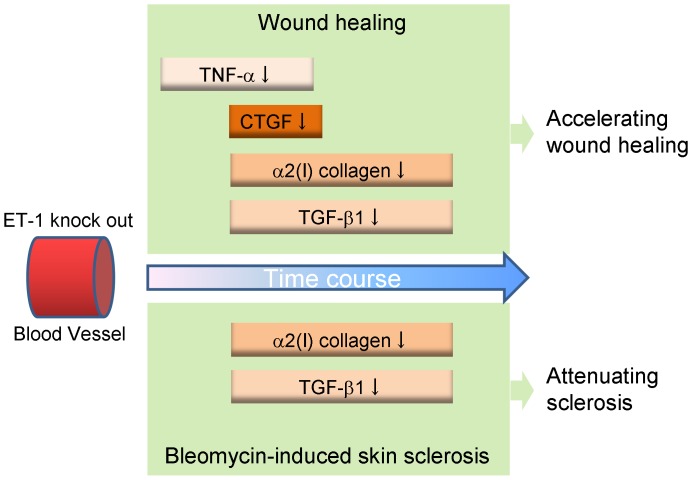
Schematic model of attenuated bleomycin-induced skin fibrosis and accelerated wound healing in endothelial cell-specific endothelin 1 knockout mice.

Based on above findings, our hypothetical model of the role of vascular endothelial ET-1 in skin fibrosis and wound healing is shown in Fig. 8. In endothelial ET-1 knockout mouse, the expression of TNF-α, CTGF, TGF-β and collagen was down-regulated. As described in Introduction, bosentan attenuates skin fibrosis and accelerates wound healing in systemic sclerosis, and such contradictory effect may be mediated by above molecules. The endothelial cell-derived ET-1 is the potent therapeutic target in fibrosis or wound healing, and investigations of the overall regulatory mechanisms of these pathological conditions by ET-1 may lead to a new therapeutic approach.

There are several limitations in this study. First, because our main focus is to investigate whether ET-1 itself, rather than its receptors, are involved in the process of fibrosis or wound heal, we did not investigate the effect of bosentan or the contribution of each receptor in our mice model. In addition, although statistically significant, data related to gene expression was not dramatic. Future studies are needed to address these points.
